# Two New M23 Peptidoglycan Hydrolases With Distinct Net Charge

**DOI:** 10.3389/fmicb.2021.719689

**Published:** 2021-09-24

**Authors:** Alicja Wysocka, Elżbieta Jagielska, Łukasz Łężniak, Izabela Sabała

**Affiliations:** International Institute of Molecular and Cell Biology in Warsaw, Warsaw, Poland

**Keywords:** peptidoglycan hydrolases, *Staphylococcus aureus*, *Staphylococcus pettenkoferi*, net charge, autolysin, bacteriocin, M23 peptidase

## Abstract

Bacterial peptidoglycan hydrolases play an essential role in cell wall metabolism during bacterial growth, division, and elongation (autolysins) or in the elimination of closely related species from the same ecological niche (bacteriocins). Most studies concerning the peptidoglycan hydrolases present in Gram-positive bacteria have focused on clinically relevant *Staphylococcus aureus* or the model organism *Bacillus subtilis*, while knowledge relating to other species remains limited. Here, we report two new peptidoglycan hydrolases from the M23 family of metallopeptidases derived from the same staphylococcal species, *Staphylococcus pettenkoferi*. They share modular architecture, significant sequence identity (60%), catalytic and binding residue conservation, and similar modes of activation, but differ in gene distribution, putative biological role, and, strikingly, in their isoelectric points (pIs). One of the peptides has a high pI, similar to that reported for all M23 peptidases evaluated to date, whereas the other displays a low pI, a unique feature among M23 peptidases. Consequently, we named them SpM23_B (Staphylococcus pettenkoferi M23 “Basic”) and SpM23_A (Staphylococcus pettenkoferi M23 “Acidic”). Using genetic and biochemical approaches, we have characterized these two novel lytic enzymes, both *in vitro* and in their physiological context. Our study presents a detailed characterization of two novel and clearly distinct peptidoglycan hydrolases to understand their role in bacterial physiology.

## Introduction

The bacterial cell wall constitutes a very complex environment, within which multiple life processes critical for bacterial survival, such as growth, division, and resistance to antibiotics, are regulated (Kuhn, 2019). Peptidoglycan, which serves as a scaffold for other components of the cell wall, comprises a highly cross-linked heteropolymer consisting of glycan and short peptides (Vollmer et al., 2008a). The precise and tightly controlled cleavage of the peptidoglycan network, which is necessary for the maintenance of its proper function, is delivered by peptidoglycan hydrolases (Vollmer et al., 2008b; Reith and Mayer, 2011; Uehara and Bernhardt, 2011). These comprise a diverse group of enzymes, including muropeptidases, amidases, carboxypeptidases, and endopeptidases. It is believed that there is at least one hydrolase for each type of bond found in peptidoglycans (Vollmer et al., 2008b).

In addition to differences in specificities, peptidoglycan hydrolases come in many flavors with regard to their modular architecture (Nelson et al., 2012; Vermassen et al., 2019). Most often, the N-terminal region plays an inhibitory role and has to be processed during enzyme maturation (Arolas et al., 2018). The resulting mature form is usually composed of an enzymatically active domain(s) (EAD) and a cell-binding domain(s) (CBD) connected by a flexible linker (Nelson et al., 2012).

Peptidoglycan hydrolases employ different mechanisms of activity regulation depending on their functions. Autolysins have to be active at precisely defined times and places during the bacterial cell cycle (e.g., the cell division ring), while the activity of bacteriocins needs to be effective and robust, while also being safe for the host. Enzymes have evolved a wide range of mechanisms to optimize their effectiveness, such as using cell wall-binding domains to find their targets (Grundling and Schneewind, 2006; Korndorfer et al., 2006).

The current study concerns two enzymes that contain an M23 peptidase domain, one of the most common catalytic domains identified among peptidoglycan hydrolases (MEROPS; Rawlings et al., 2008; Mitchell et al., 2021). Enzymes from this family are needed during cell growth and division (autolysins, such as LytM; Ramadurai and Jayaswal, 1997); they are used as weapons against other bacteria that occupy the same ecological niche [e.g., lysostaphin (Lss)] (Thumm and Gotz, 1997) or play a role in bacterial virulence (e.g., LasA; Spencer et al., 2010). M23 peptidases typically display specificity toward interpeptide bridges of various bacterial peptidoglycans. Several hydrolases with such a catalytic domain have been characterized to date, including Lss, LytM, and LytU, that predominantly cleave glycine–glycine bonds in peptidoglycan cross-bridges of *Staphylococcus* spp. (Schindler and Schuhardt, 1964; Sabala et al., 2012; Raulinaitis et al., 2017); zoocinA or EnpA that cleave D-alanine–L-alanine bonds in peptidoglycan cross-links present in *Streptococcus* and *Enterococcus* spp. (de Roca et al., 2010; Lester and Simmonds, 2012); and LasA, which has a broader range of cleavable substrates (glycine–glycine/alanine/phenylalanine and tyrosine/leucine; Spencer et al., 2010).

Here, we report two novel peptidoglycan hydrolases belonging to the M23B family (MEROPS; Rawlings et al., 2008) that were identified in a recently described *Staphylococcus* sp. (Trulzsch et al., 2002). Importantly, although they share approximately 60% amino acid sequence identity, their isoelectric points (pIs) are strikingly different. We have analyzed their genetic background, the frequency of their gene occurrence, and their localization in the host cell. Based on the results and their biochemical features, we discuss the possible roles that these two peptidoglycan hydrolases, which are clearly distinct from those so far described, play in bacterial cell metabolism.

## Materials and Methods

### Bacterial Strains and Growth Conditions

Bacterial strains were purchased from the Polish Collection of Microorganisms (PCM, Institute of Immunology and Experimental Therapy, Wrocław, Poland), the German Collection of Microorganisms and Cell Cultures (DSMZ), American Type Culture Collection (ATCC), or the Czech Collection of Microorganisms (CCM, Masaryk University, Brno, Czech Republic). Others kindly provided by the institutions are listed in the Supplementary Table 1.

*Escherichia coli* cells were cultured in lysogeny broth (LB) at 37°C with shaking (250rpm). All other strains were cultured in tryptic soy broth (TSB) at 37°C with shaking (80rpm).

### Molecular Biology Methods

The *spm23_A* gene was amplified from crude genomic DNA (gDNA) isolated from the *S. pettenkoferi* DSM 19554 strain following lysostaphin treatment. The *spm23_B* fragment containing the mature form of its protein product was synthesized by Bio Basic (Markham, Ontario, Canada). Both fragments and separate domains were cloned into the pET22b(+) expression vector (Merck, Darmstadt, Germany). To generate CBD/green fluorescent protein (GFP) fusions (C-terminus), each of the CBDs was cloned into the pWALDO expression vector (Waldo et al., 1999). The three CBD_A variants were obtained by site-directed mutagenesis. All the generated constructs were confirmed by sequencing (Eurofins Genomics, Ebersberg, Germany). Basic information about the constructs and the parameters of the purified proteins, calculated using the ProtParam bioinformatic tool (Artimo et al., 2012), is listed in Supplementary Table 2. Screening for the presence of *spm23_A* and *spm23_B* genes in the genomes of the tested bacterial strains was performed using crude gDNA extraction with lysostaphin treatment and the same primers and reaction conditions as those used for the generation of the above-described vectors.

### Protein Expression and Purification

*Escherichia coli* BL21 (DE3) cells were transformed, plated on LB agar supplemented with the appropriate antibiotic selection marker, and a single colony was inoculated in a small volume of overnight culture in LB at 37°C with shaking (250rpm). The next day, the inoculum was refreshed (1%) and the cells were cultured in the same conditions to an OD_600_ of 0.6–0.8 following the addition of 1mM isopropyl β-D-1-thiogalactopyranoside (IPTG) for induction, the temperature was decreased to 18°C and protein production was continued overnight. The bacterial pellet containing overexpressed protein was suspended in Buffer A (20mM Tris-HCl pH 7.0, 1M NaCl), and the cells were sonicated. The lysate was clarified by centrifugation at 95,834×*g*, and the supernatant was dialyzed overnight in Buffer B (20mM Tris-HCl pH 7.0, 80mM NaCl). The next day, the SpM23_A and SpM23_B dialyzed lysate was filtered and loaded onto WB40S resin (Bio-Works, Uppsala, Sweden) and the fractions corresponding to the peaks were eluted with a gradient of Buffer A in 10 column volumes (CVs). CBD_A was loaded onto DEAE-Sepharose resin (Cytiva, Marlborough, Massachusetts, United States), and the flow-through fraction containing the recombinant enzyme was collected. The His-tagged proteins were loaded onto HisTrap columns (Ni Sepharose High-Performance HisTrap HP, Cytiva, Marlborough, United States, Chicago, Illinois, United States) equilibrated in buffer containing 20mM Tris-HCl pH 7.0, 1M NaCl, 15mM imidazole, and the sample was eluted with the buffer containing 20mM Tris-HCl pH 7.0, 0.5M NaCl and 300mM imidazole in 20 CVs. Protein fractions were pooled and concentrated on Amicon 3,000 or 10,000 NMWL centrifugal filters. All the protein samples were subjected to size-exclusion chromatography in 20mM Tris-HCl pH 7.0 buffer containing 200mM NaCl and 10% glycerol on a Superdex75 16/600 (GE Healthcare) column using the ÄKTA Purifier system. The purity of Coomassie blue-stained recombinant enzymes was assessed by SDS-PAGE. The peak fractions were concentrated on Amicon centrifugal filters and used for activity assays. For long-term storage, the proteins were flash-frozen in liquid nitrogen and stored at −80°C. Lss was purified as previously described (Jagielska et al., 2016).

### Cellular Fraction Preparation

The OD_600_ of overnight cultures of *Staphylococcus* spp. was adjusted to ~0.8, and an equal volume of each cell culture (50ml) was collected following centrifugation. The extracellular fraction was collected and concentrated approximately 25-fold on Amicon centrifugal filters (10,000 NMWL). LiCl was used to extract the proteins attached to the cell wall by ionic interactions (cell wall protein fraction; Mani et al., 1993). Briefly, cells were washed once with 1ml of ice-cold Milli-Q water, resuspended in 0.5ml of 3M LiCl, and incubated for 30min at 4°C on a rotating wheel. The cell wall was pelleted by centrifugation, and the supernatant was collected. The remaining insoluble fraction was incubated with 0.5ml of PBS supplemented with 2% SDS for 30min at 37°C with agitation (150rpm) to collect the cell membrane and cytoplasmic protein fraction. The remaining insoluble cell wall was pelleted, and the supernatant was collected. All the samples were stored at −20°C until used.

### Western Blot

Cellular fractions were subjected to Western blot analysis. Briefly, 20μl/well of each sample was separated by 10% tricine SDS-PAGE. For the positive controls, 20μg of each recombinant enzyme was used. Samples from gels were transferred onto PVDF membranes (semi-dry transfer, 25min, 1 A, 25V), blocked with 5% milk in TBS buffer containing Tween 20 (TBST), incubated overnight with antibody against lysostaphin (α-M23+SH3b, dilution 1:1,000, Cat. no. PAb102, AIC Biotech), extensively washed, and finally incubated with a secondary antibody (anti-rabbit, Cat. no. 31460, Thermo Scientific). The results were visualized using enhanced chemiluminescence and imaged using the ChemiDoc system (Bio-Rad, United States). Membranes were then stripped by boiling for 5min in 50mM EDTA pH 8.0, followed by thorough washing in TBS-T. When no signal was detected, the membrane was blocked and incubated with the primary antibody, α-M23 (diluted 1:200), prepared as previously described (Sabala et al., 2012). The following procedure was the same as that described for the α-M23+SH3b antibody.

### Charge Analysis

#### 2D Electrophoresis

The procedure was performed using components of ReadyPrep 2-D Starter Kit (cat. 163-2105, Bio-Rad) accordingly to the manufacturer protocol. Firstly, the samples were desalted (final ~10mM) upon dialysis to low-salt buffer and concentration on Amicon^™^ centrifugal filters. Next, samples were diluted in rehydration buffer up to 4-fold and loaded on the ReadyStrip^™^ IPG Strips 3-10 (cat. 163-2000, Bio-Rad). After overnight rehydratation, the strips were run on the PROTEAN IEF (Bio-Rad) using the protocol suitable for 7cm-long strip. When the run was completed, the gel was washed with the provided equilibration solutions, briefly washed in the cathode buffer, and transferred to 10% SDS-PAGE gel without the stacking part. ReadyPrep^™^ Overlay Agarose (163-2111) was poured on the top, and after solidification, the gel was run using standard parameters and imaged with Coomassie blue staining.

#### Cytochrome C

The cytochrome C (Cyt C) assay was performed as previously described (Peschel et al., 1999). Briefly, a single colony of each bacterial strain was inoculated in 20ml of TSB and cultivated at 37°C with shaking at 100rpm. The next day, the culture was pelleted and resuspended in reaction buffer (20mM ammonium acetate, pH 4.6). The cell density of each strain was adjusted to the same value (OD_600_ ~30), and 600μl of each bacterial suspension was pelleted. The pellets were resuspended in 0.2mg/ml Cyt C solution in reaction buffer and incubated for 15min at 37°C. The sample was subsequently pelleted, and the absorbance of the solution was immediately measured at 410nM using an Infinite F50 microplate reader (TECAN, Switzerland). Measurements were performed in a 96-well plate at room temperature. The results were compared with those of the negative control (the Cyt C sample without bacteria). All the experiments were performed at least three times in triplicate.

### Activity Assays

#### Turbidity Reduction Assay

A single colony of each bacterial strain was inoculated in a small volume of overnight culture in TSB and cultivated at 37°C with agitation (80rpm). The next day, fresh TSB (2%) was added and the bacteria were cultured to the mid-exponential growth phase (OD_600_ 0.6–0.8). The cultures were then pelleted and resuspended in lysis buffer (50mM glycine-NaOH, pH 8.0). For conditions of high ionic strength, the buffer was supplemented with 100mM NaCl. The cell density was adjusted to approximately 1×10^8^ colony-forming units (CFUs)/ml (OD_600_ ~1). The enzymes were added to a final concentration of 100nM, and turbidity reduction was monitored every 10min at the 620-nM wavelength using the Infinite F50 microplate reader. The experiment was performed in a 96-well plate at room temperature for 1h. The results were compared with those of the negative control (the bacterial cell suspension without added enzyme). All experiments were performed at least three times in triplicate.

#### Spot Assay

*Staphylococcus aureus* NCTC 8325-4 culture and its suspension in reaction buffer (50mM glycine-NaOH, pH 8.0) were prepared as described above. Each enzyme (500nM final concentration) was added, and the decrease in the OD_600_ was monitored every 10min using the Infinite F50 microplate reader. The reaction was duplicated in another 96-well plate and served for the live cell count. At selected time points (10, 20, and 60min), 40μl of each sample was discarded and 1mM EDTA, a metalloprotease inhibitor, was added to stop the reaction. Next, each sample was subjected to 10-fold serial dilutions and 5μl was plated in rows on TSB-A plates. The plates were incubated overnight at 37°C, and the results were documented the next day. Each sample was plated in triplicate, and the experiment was independently performed three times.

#### Bioactivity Assays

Bacterial lawns were prepared as described above. Harvested cells were resuspended in PBS, pH 7.2, and the OD_600_ was adjusted to 1. A 1-ml aliquot was subsequently spotted on TSB-A plates (susceptible lawn variant) or first mixed with the recombinant CBD_A domain and then spotted (30μM final concentration; resistant lawn variant). The suspension was evenly distributed over the plate and left to dry under a sterile hood. Then, 0.5ml of the *S. pettenkoferi* culture was centrifuged, the culture medium was discarded, and the obtained pellet was resuspended in 20μl of PBS. An equal volume of this suspension was spotted on each lawn variant. As a positive control, 20μl of recombinant enzyme (400nM in PBS) was spotted on a filter paper disk placed directly on each lawn. As a negative control, 20μl of PBS buffer was spotted on a filter disk paper placed on each lawn. The plate was incubated at 37°C overnight, and the results were documented the next day.

#### Binding Assays

Binding assays were performed based on a previously described protocol (Grundling and Schneewind, 2006). Briefly, bacterial cells prepared as described above were resuspended in PBS, pH 7.2, and the OD_600_ was adjusted to ~20. A 130-μl aliquot of bacterial cell suspension was mixed with each CBD fused to GFP (CBD-GFP) at a final concentration of 1μM, incubated at room temperature for 15min, and then pelleted. The negative controls (A: bacteria without the addition of the enzyme; B: each CBD-GFP domain in 150μl of the PBS buffer alone; and C: GFP without the addition of bacteria) were also included. The supernatant was treated as the unbound fraction, and the fluorescence intensity was measured in a 96-well microplate reader (excitation: 488nM, emission: 508nM; Infinite M1000, TECAN). Values were expressed relative to negative control B and, together with the possible emission (A) and unspecific binding (C) values, were used to calculate the % value of the bound fraction. All the experiments were performed at least three times with three replicates for each sample.

## Results

### The Modular Architecture of the Novel M23 Peptidases

A BLAST search with the sequence of mature Lss from *S. simulans* comprising the catalytic and cell wall-binding domains (the region located between residues 251 and 493, GenBank ID WP_013012297.1) resulted in the identification of two sequences from *S. pettenkoferi* VCU 012. This is a relatively novel species of coagulase-negative staphylococci first isolated from clinical specimens (Trulzsch et al., 2002). Further analysis by Mansson and colleagues revealed a close genomic relatedness between clinical and environmental isolates, even though differences within their gDNA differentiate them into two major clades (Mansson et al., 2017). The pathogenic potential of *S. pettenkoferi* is likely to be low, and their identification in clinical samples can, in most cases, be regarded as a contamination of the skin, which most likely constitutes the natural habitat of this species (Mansson et al., 2017).

**Figure 1:**
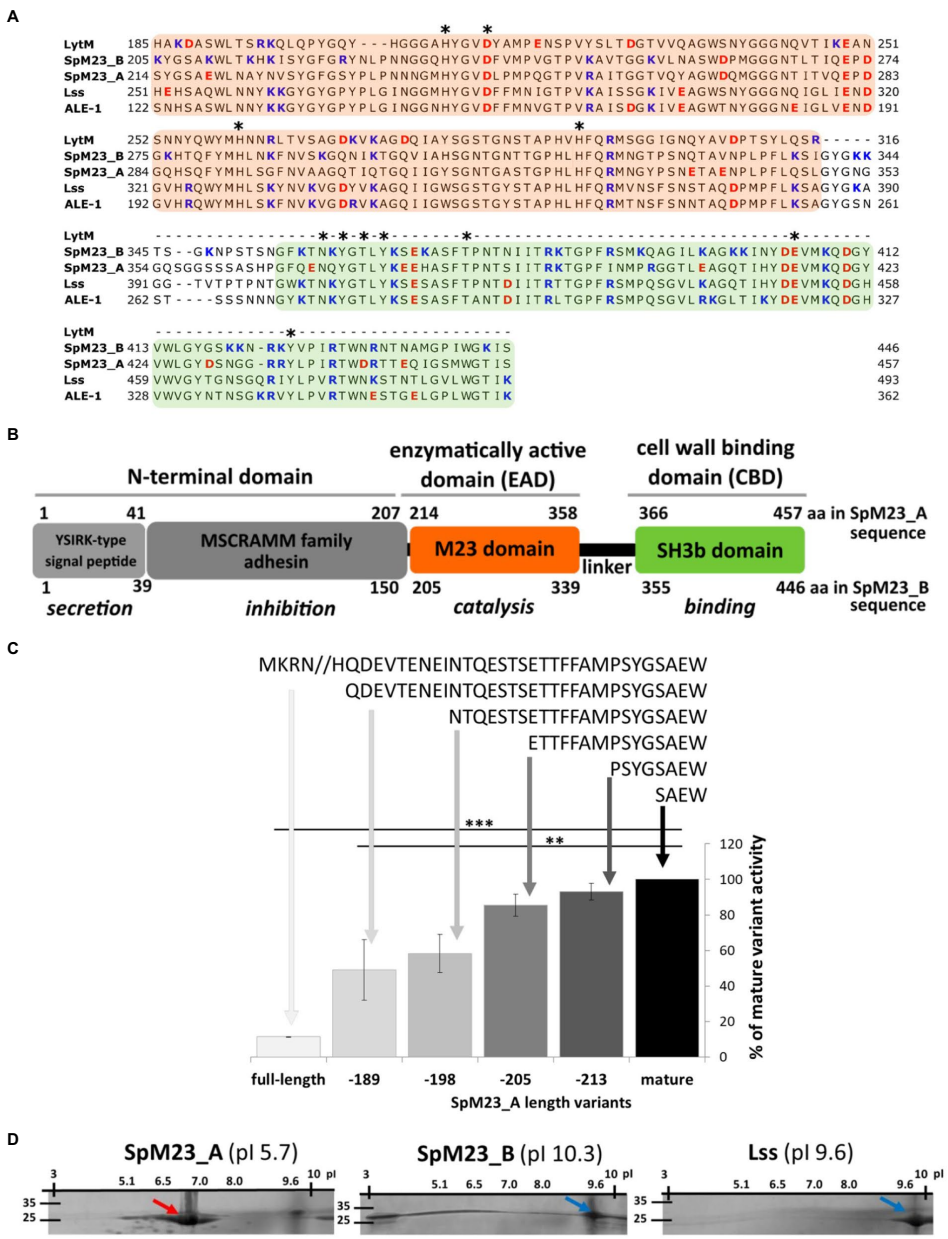
Modular architecture and charge analysis of the novel M23 peptidases. **(A)** Sequence alignment of the selected enzymes with the M23 catalytic and SH3b binding domains in CLUSTAL Omega (Madeira et al., 2019). Domain sequences are indicated against a background colored according to the color scheme in **(B)**. The conserved amino acids involved in catalysis or binding are indicated with an asterisk. Negatively charged amino acids are marked in red and positively charged amino acids in blue. Sequence GenBank ID: Lss WP_013012297.1; Ale_1 AAC12656.1; LytM PAG39821.1; SpM23_A WP_002472647.1; SpM23_B ASE36562.1A. **(B)** Schematic representation of the domain organization of the SpM23_A and SpM23_B enzymes. The domain boundaries were set according to the divisions presented in GenBank and the Conserved Domains Database (Lu et al., 2020). Function prediction based on the amino acid sequence and motif conservation is indicated in italics. **(C)** The lytic activity of full-length SpM23_A and that of its shortened N-terminus derivatives. The recombinant enzymes were named according to the number of amino acids removed from the N-terminal domain, colored and ranked according to the length of the amino acid sequence from the longest (pale grey) to the shortest amino acid chain (black). The activity of each enzyme was measured using a turbidity reduction assay in 1h at room temperature in 50mM glycine, pH 8.0 against *Staphylococcus aureus* RN4220 ΔtagO using 100nM enzyme concentrations. The chart presents the relative bacteriolytic activity of the generated enzymes normalized to that of the most active one, namely, the shortest derivative of full-length SpM23_A. The decrease in the turbidity of the bacterial suspension in the reaction buffer without the addition of the lytic enzyme served as a negative control, and the values were subtracted from the presented results. The bars show the standard deviation calculated based on the results from at least two independent experiments. Asterisks indicate the level of statistical significance between mature enzyme and each sequence variant (*t*-test for two independent means); ^*^p<0.05; ^**^p<0.001. **(D)** Documentation of 2D electrophoresis assay (isoelectrofocusing combined with 10% SDS-PAGE gel) shows the migration of recombinant proteins accordingly to their pI. The theoretical pI calculated for each enzyme was indicated in parenthesis. The arrows, color coded accordingly to the predicted pI (red for acidic and blue for basic proteins), indicate the point at which most of the protein sample has stopped migrating.

These two novel hydrolases, SpM23_A (GenBank ID: WP_049408311.1) and SpM23_B (GenBank ID: ASE36562.1), share 65% amino acid identity and 80% similarity and exhibit active center conservation as well as high sequence similarity with Lss (Figure 1A; Supplementary Table 3). Sequence analysis revealed the presence of several elements already identified in Lss and other peptidoglycan hydrolases.

#### Signal Peptide

Using the SignalP-5.0 server (Almagro Armenteros et al., 2019), we identified a signal peptide at the N-terminus of both SpM23_A (41 residues) and SpM23_B (39 residues; Figure 1B). Both are predicted to be directed to the general Sec/SPI secretory pathway (predicted likelihood: 0.56 and 0.97, respectively), although SpM23_A is also predicted to share some similarity with lipoprotein signal peptide (Sec/SPII, likelihood: 0.40) and contains C28 cleavage site predicted to be recognized by lipoprotein Signal peptidase II. This was further supported by the identification of a transmembrane domain between residues 12 and 33 (Phobious; Nielsen et al., 2019) and a membrane lipoprotein lipid attachment site (ScanProsite; de Castro et al., 2006; Supplementary Figure 1), hinting that SpM23_A may undergo transport to the cell membrane.

Both signal peptides have been classified as YSIRKG/S-type, a sequence that is known to target the hybrid protein to ring-like structures formed at the site of cell division, the so-called cross-wall (DeDent et al., 2008). The LPXTG motif that is recognized by sortase and used as an anchor for cell wall proteins, commonly found in YSIRKG/S-type signal peptides (Bae and Schneewind, 2003), was not identified in the sequence of either SpM23_A or SpM23_B. Both enzymes are classified as extracellular based on the PSORT algorithm, although SpM23_B has a slightly higher score than SpM23_A, indicative of cell wall localization (Yu et al., 2010).

#### Propeptide

A Conserved Domains Database search (Lu et al., 2020) showed that the region between residues 4 and 207 in SpM23_A and between residues 8 and 150 in SpM23_B displays sequence similarities to the MSCRAMM family of bacterial adhesins (E-value: 4.87e-07; Figure 1B); however, the identified similarity was found to be limited to a short N-terminal segment (WP_089431353.1; query cover: 5 and 8%, identity: 29 and 34%, respectively). The RADAR tool (Madeira et al., 2019) identified two 70-amino-acid-long repeated sequences in the SpM23_A N-terminal domain and three 30-amino-acid-long repeated sequences in that of SpM23_B (Supplementary Figure 1).

The propeptides of proteolytic enzymes often play an inhibitory role (Tang et al., 2003; Boon et al., 2020). This is also the case for SpM23_A, the full-length form of which displays strongly diminished lytic activity against *S. aureus*. We generated a series of N-terminally truncated derivatives of SpM23_A and tested their lytic activity. The lowest activity was observed for the longest construct, and the highest for the shortest construct (Figure 1C). Although we did not have the full-length SpM23_B recombinant protein to confirm whether it displays the same mechanism of activation, the mature form of this enzyme exhibited very high activity. The lytic assay (Supplementary Figure 2A) results revealed that a 500-nM concentration of each mature enzyme eradicated up to 1×10^8^ CFUs/ml of *S. aureus* in 1h at room temperature. Notably, SpM23_B and lysostaphin were able to eliminate bacteria faster than SpM23_A (1×10^8^ CFUs/ml in 10min vs. no observed activity at this time point, respectively).

Combined, these results support that the N-terminal domains of SpM23_A and SpM23_B are involved in the inhibition of enzyme activity as has also been described for Lss and LytM (Thumm and Gotz, 1997; Odintsov et al., 2004), and may also need to be proteolytically removed for the activation of peptidase activity (Arolas et al., 2018). Overall, our analysis justifies classifying the full-length forms of both SpM23_A and SpM23_B as prepropeptides.

#### The M23 Catalytic Domain

The catalytic domains (or EADs) of the two enzymes display several features characteristic of M23 peptidases. They both contain the conserved motifs **H**XXX**D** and HX**H** that comprise zinc ligands, i.e., two histidines and one aspartic acid residue (bold text). We confirmed the presence of zinc atoms in both proteins by atomic spectroscopy (data not shown). Other amino acids that are conserved in the M23 family of metallopeptidases are also found in the SpM23_A and SpM23_B protein sequences. These include two histidines (SpM23_A: H290 and H323; SpM23_B: H281 and H314) proposed to act as general bases for LytM (Grabowska et al., 2015), Lss (Sabala et al., 2014), and LasA (Spencer et al., 2010); and tyrosine/arginine residues, such as LytM Y204, which are reported to help stabilize substrate transition states (Grabowska et al., 2015; SpM23_A Y233, SpM23_B Y224).

Overall, the above features, as well as high amino acid sequence identity, classify the catalytic domains of SpM23_A and SpM23_B as new members of the MEROPS M23 family of zinc-dependent metallopeptidases.

#### The SH3b Cell Wall-Binding Domain

The catalytic domains of both SpM23_A and SpM23_B are linked to their respective C-terminal CBDs by 15–17-amino-acid-long peptides. Based on amino acid homology, these CBDs are classified as members of the SH3b family found in various peptidoglycan hydrolases (Becker et al., 2009b). SH3b family members have been shown to play a crucial role in the recognition and binding of bacterial cell walls (Baba and Schneewind, 1996; Grundling and Schneewind, 2006; Lu et al., 2006). In the SpM23_A and SpM23_B enzymes, the CBDs display, respectively, 67 and 70% amino acid identity with the CBD of Lss (Becker et al., 2009b) and 63 and 66% amino acid identity with that of Ale-1 from *S. capitis* (Lu et al., 2006). We observed that, as occurs with the CBD of lysostaphin, both CBD_A and CBD_B recognize and bind *S. aureus* cells (Supplementary Figure 2B).

#### Charge Analysis

Despite sharing very high sequence identity/similarity, SpM23_A and SpM23_B have substantially different pIs. The pIs of SpM23_A and SpM23_B were calculated as 5.68 and 10.28, respectively (hence the names SpM23_**A** for “**A**cidic” and SpM23_**B** for “**B**asic”). We verified it experimentally using 2D electrophoresis that SpM23_B migrates as basic protein (around 9.7) and the SpM23_A as mildly acidic (around 6.8) (Figure 1D). Although the experimentally defined pI of SpM23_A is higher and the SpM23_B lower than the predicted values, the 2D electrophoresis confirmed that the SpM23_A and SpM23_B enzymes differ in terms of their net charge.

The negative surface charge of SpM23_A is unique among peptidases as almost all members of this family described to date are basic, including Lss (Schindler and Schuhardt, 1964), LasA (Kessler et al., 1993), and Ale-1 (Sugai et al., 1997a). Although charged amino acids are found on both domains, most of them are located on the CBD (65–68%).

### Genetic Context of the *spm23_A* and *spm23_B* Genes

#### *spm23_A* Is More Widely Distributed Than *spm23_B*

**Figure 2:**
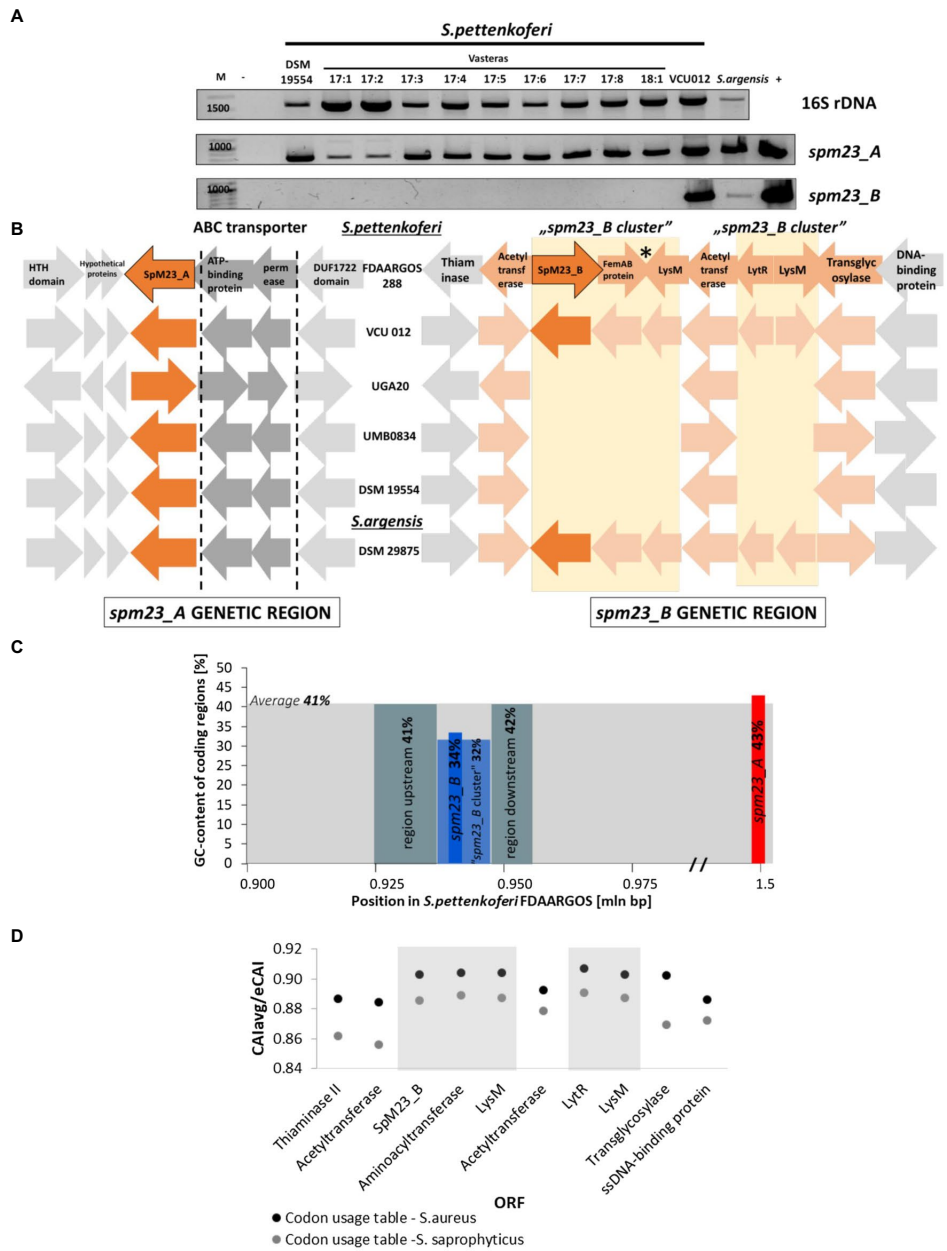
The genetic context of the *spm23_A* and *spm23_B* genes. **(A)** The PCR screening of *Staphylococcus pettenkoferi* strains for the presence of the *spm23_A* and *spm23_B* genes. The reaction targeting the 16S rRNA gene was treated as an internal control of the genome DNA template quality. −, reaction without added DNA; +, reaction with the addition of plasmid encoding each gene. **(B)** Schematic representation of the genetic neighborhood of the *spm23_A* and *spm23_B* genes in the selected strains. The left panel shows the gene arrangement of *spm23_A* and the right panel the gene arrangement of *spm23_B*. The pale-yellow square indicates part of the “*spm23_B* cluster.” Arrows in shades of orange indicate genes involved in envelope metabolism, an asterisk indicates the site where inverted repeats were identified. The direction of the arrow corresponds to the gene orientation. HTH domain: HTH domain-containing protein; LysM: LysM peptidoglycan binding domain-containing protein. The GenBank ID numbers for the genetic region of SpM23_A are as follows: HTH-domain (QQC38290.1), hypothetical proteins (QQC38291.1, QQC38292.1, and QQC38293.1), ABC transporter ATP-binding protein (QQC38295.1), ABC transporter permease (QQC38296.1), and DUF1722 domain (QQC38474.1). For the SpM23_B genetic region, the ID numbers are provided in Supplementary Table 5. **(C)** The GC-content of the selected genetic regions studied in the *S. pettenkoferi* genome (GenBank ID: CP066062.1). The *spm23_A* genetic region is marked in red, *spm23_B* in blue, the peptidoglycan metabolism-related fragment in the neighborhood of *spm23_B* in pale blue, and the regions ~200bp up- and downstream of *spm23_B* in grey. **(D)** Codon adaptation index (CAI) analysis using the codon usage table for *S. aureus* NCTC 8325 (black dots) and *S. saprophyticus* (grey dots; Puigbo et al., 2008). The “*m23_b* cluster” is indicated against a grey background. CAIavg, codon adaptation index averaged; eCAI, estimated CAI. The name abbreviations are the same as in panel **B**.

To verify the presence of the *spm23_A* and *spm23_B* genes among *S. pettenkoferi* strains, we collected 11 isolates representative of this species from different sources (Supplementary Table 1) and screened their genomes for the presence of *spm23_A* and *spm23_B*. The *spm23_A* gene was detected in all isolates tested using PCR with primers targeting the genetic locus between 1,549,677 and 1,551,050bp (GenBank ID: CP022096.2). In contrast, using primers targeting the *spm23_B* gene in the region between 934,367 and 935,707bp within the same genomes, a product of the correct size was identified in only one strain of *S. pettenkoferi*, namely, VCU 012 (Figure 2A).

We also searched GenBank for the presence of these genes in available bacterial genome sequences. The *spm23_A* gene was found in all 18 deposited *S. pettenkoferi* sequences, whereas *spm23_B* was identified in only two (ASE36562.1 and EHM72108.1). Interestingly, both genes were also found in *Staphylococcus argensis* (strain DSM 29875), which is closely related to *S. pettenkoferi* (Heß et al., 2015). We confirmed the presence of both genes in this strain by PCR.

#### SpM23_B Forms Part of a Genetic Cluster Related in Function to Peptidoglycan Metabolism

The differences in the occurrence of the investigated genes among *S. pettenkoferi* strains prompted us to study their genetic neighborhood and look for hallmarks of their origin in the genome. This information could also provide some hints as to the potential role of both SpM23_A and SpM23_B.

The genetic neighborhood of the *spm23_A* gene is very well conserved in all the analyzed strains. In contrast to its genetic neighbors, this gene is not been predicted to form an operon (Operon-mapper; Taboada et al., 2018). Two hypothetical protein-coding genes that form the operon were found in close proximity. One of the putative proteins contains a predicted signal peptide and is described as a membrane lipoprotein lipid attachment site-containing protein. An operon of the ABC transporter, a transmembrane trafficking system involved in antimicrobial peptide transport and/or lipoprotein export (Lu et al., 2020), is found downstream of the *spm23_A* gene. We therefore concluded that *spm23_A* is located in a conserved genetic environment containing open reading frames (ORFs) that are functionally related to peptide/protein secretion.

The *spm23_B* gene was found to be present in the sequences of 3 genomes deposited in the databases and was accompanied by five ORFs in each case (Figure 2B). According to Operon-mapper, these genes do not form an operon (Taboada et al., 2018). The five ORFs were not found in the genomic sequences of strains that harbor *spm23_A* but not *spm23_B*; thus, we concluded that *spm23_B* was inherited together with the five ORFs, and called it the “*spm23_B* cluster.”

*spm23_B* is located near a gene encoding an aminoacyl transferase that displays similarity to FmhA, which is reported to be involved in the incorporation of serine residues into *S. aureus* peptidoglycan cross-bridges (Willing et al., 2020), as well as genes encoding the Epr and Lif proteins, known to provide resistance to Ale-1 and Lss in *S. capitis* and *S. simulans*, respectively (Sugai et al., 1997b; Thumm and Gotz, 1997; Supplementary Table 4).

The “*spm23_B* cluster” (Supplementary Table 5) also contains duplications, namely, the pair of the acetyltransferases (amino acid sequence identity: 66%) and the LysM peptidoglycan binding domain-containing proteins (identity: 98%). The function of these genes has been assigned to cell wall metabolism; acetyltransferases transfer *O*-acetylate groups to carbohydrate moieties (Slauch et al., 1996), and lytic enzymes containing the LysM binding motif are known to bind the glycan part of peptidoglycan (Buist et al., 2008). One of the enzymes containing LysM binding motif encodes a chitinase domain with specificity narrowed to the β-1,4-linkage of polysaccharides (Conserved Domains Database; Lu et al., 2020) and another a CHAP domain with endopeptidase and amidase activities (Becker et al., 2009a). Overall, the “*spm23_B* cluster” comprises a set of genes functionally related to cell wall metabolism.

To identify the origin of *spm23_A* and *spm23_B*, we first investigated their GC content relative to all the coding regions of the *S. pettenkoferi* genome (GenBank ID: CP066062.1). Although the GC content did not differ for *spm23_A*, *spm23_B*, together with its genetic cluster, displayed a lower GC content compared with that for *spm23_A* as well as with the average for all the coding regions (Figure 2C). Moreover, the “*spm23_B* cluster” displayed a marginally higher codon adaptation index (CAI; Puigbo et al., 2008) compared with the codon usage for reference *S. aureus* NCTC 8325 and *S. saprophyticus*, close relatives of *S. pettenkoferi* (Trulzsch et al., 2002; Figure 2D). These differences in GC content and CAI between the *spm23_B* cluster and the surrounding regions suggest a distinct genetic origin for this DNA fragment.

We also checked for the presence of hallmarks of horizontal gene transfer (HGT). The nearest identified prophage gene cluster to *spm23_A* (phage region: 1,409,507–1,424,957; *spm23_A* locus: 1,549,677–1,551,050) is probably too far to be implicated in the transduction of this gene (PHAST; Zhou et al., 2011). According to the Integrated Microbial Genomes & Microbiomes database,^1^ no sequence of HGT origin was found in the neighborhood of the “*spm23_B* cluster” and no significant hits for insertion sequences were identified inside the cluster or in its proximity (200-bp region up- and downstream; ISFinder; Zhang et al., 2000). A pair of 21-bp inverted repeats (IRs) were found within the “*spm23_B* cluster” upstream of the *lysM* gene (Figure 2B), indicative of the occurrence of DNA rearrangements, such as inversions or duplications (Bi and Liu, 1996; Lin et al., 2001).

Given the genetic differences and the large genetic distance between the *spm23_A and spm23_B* genes, it is unlikely that the activity of these novel hydrolases is not coregulated *in vivo*. We did not observe any additive effect of these two enzymes when tested *in vitro* (data not shown).

#### Sequence Differences of SpM23_A Among Closely Related Strains

The region comprising the mature SpM23_A enzyme is very well conserved among *S. pettenkoferi* strains (EAD_A: 99–100% identity, 100% similarity; CBD_A: 96–97% identity, 98–100% similarity among the deposited GenBank sequences). The only differences were found in a short stretch of four amino acid residues grouped at the C-terminus of CBD_A (Supplementary Figure 3A). All these residues are located in the extra loop, and small β-sheet and their side chains are surface-exposed (positions 443, 445, 447, and 449; Supplementary Figure 3B). The first two positions are occupied by polar or negative residues (N/D and N/T), the third is always negatively charged (D/E), and the fourth is hydrophobic (M/I). Although the conservation of this region is not high among other SH3b domains and the region is far from the binding groove, the corresponding region of Ale-1 was proposed to be involved in its interaction with peptidoglycan components other than cross-bridges (Lu et al., 2006). We investigated if such naturally occurring variants play a role in the performance of CBD_A by comparing the binding activity of all the CBD_A sequence variants fused to GFP (Supplementary Figure 3C). As already noted (Supplementary Figure 2B), CBD_A binds *S. aureus*. In further optimization trials, we observed that CBD_A displayed the highest binding efficiency toward a mutant lacking teichoic acids, namely, *S. aureus* RN4220 ΔtagO (Supplementary Figure 4). Accordingly, this mutant was selected to identify the most prominent differences among the CBD_A sequence variants. The results of the binding assays demonstrated that, among all the variants, only the least abundant (D-T-E-I) exhibited decreased binding efficiency, underlying the relevance of the three residues, namely, N445, D447, and/or M449, present in the distal loop, for substrate recognition/binding.

### The Catalytic and Binding Specificity of SpM23_A and SpM23_B

#### The Pattern of Susceptibility Toward SpM23_A and SpM23_B Differs Among Closely Related Strains

Our preliminary results demonstrated that the mature forms of both enzymes can lyse *S. aureus* cells. Given that all identified *S. pettenkoferi* strains carry one or the other of the studied genes, we wondered whether these strains are resistant to the hydrolases they produce.

**Figure 3:**
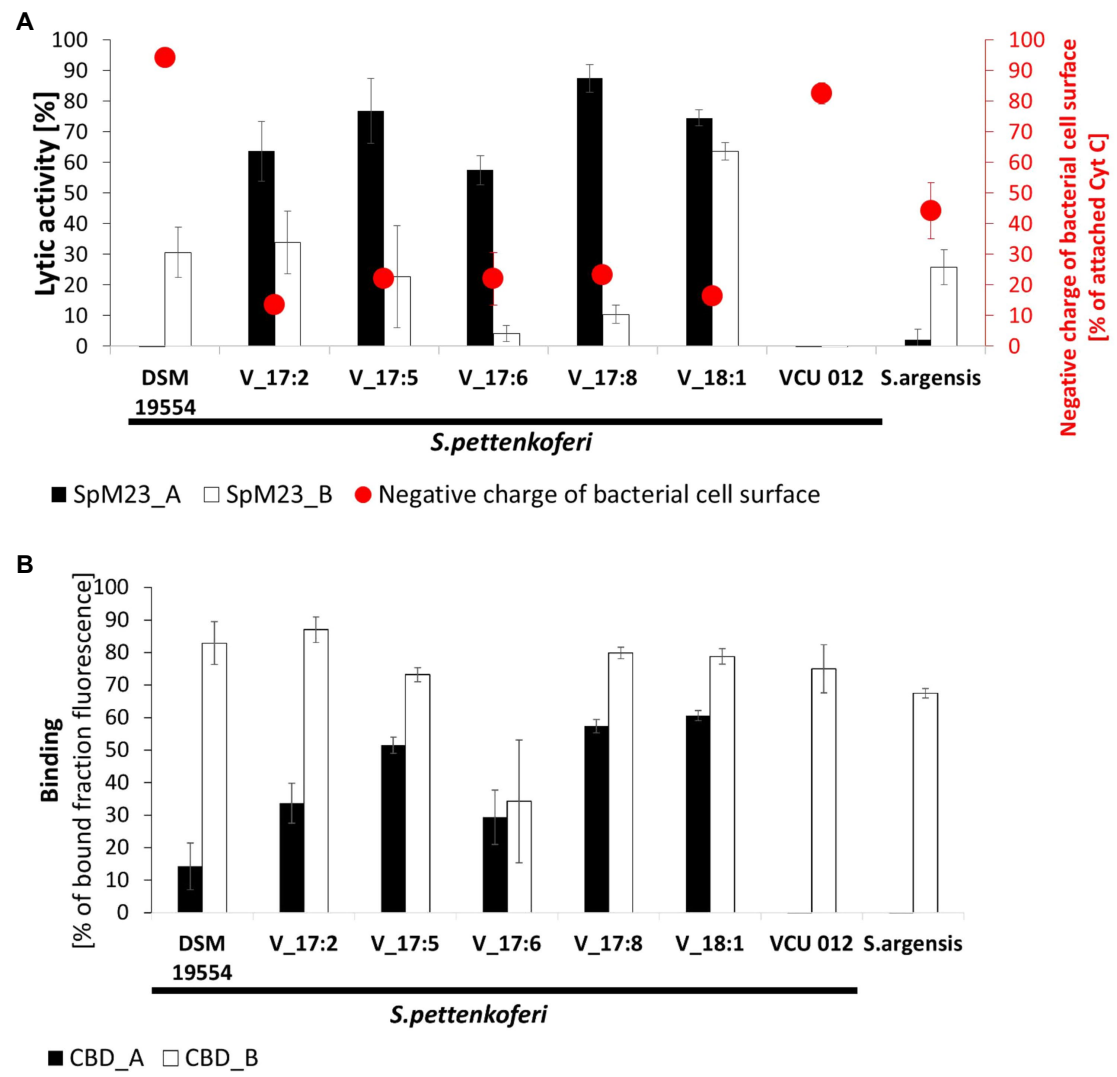
The lytic and binding specificities of SpM23_A and SpM23_B toward bacterial strains that harbor their respective coding genes. **(A)** Comparison of the lytic activity and the net charge on the surface of *Staphylococcus pettenkoferi* strain cells. The net charge was estimated by cytochrome c (Cyt C) assay performed in ammonium acetate buffer, pH 4.6. Lytic activity was measured based on decreases in OD_600_ values after 1h of reaction. Bacteria resuspended in reaction buffer without the addition of the lytic enzyme served as the negative control; the reductions in OD_600_ values in this sample were subtracted from the presented values. The assay was performed in 50mM glycine buffer, pH 8.0. All the experiments were performed three times. **(B)** A binding assay was performed on a set of *S. pettenkoferi* strains and *S. argensis* to compare the binding efficiencies of recombinant CBD_A and CBD_B fused to GFP. The fluorescence of the negative control (GFP alone) was subtracted from the presented results. The assay was performed in PBS, pH 7.2, at room temperature. Fluorescence was measured with excitation at the 488nM wavelength and emission at the 508nM wavelength.

The results of the lytic assays (Figure 3A) revealed that the susceptibility toward either SpM23_A or SpM23_B among each set of closely related strains is very diverse. None of the enzymes was active against strain VCU 012 of *S. pettenkoferi*, which carries both genes; however, SpM23_B could lyse cells of *S. pettenkoferi* strain DSM 19554, which carries only the *spm23_A* gene. In contrast, none of the Vasteras isolates that carry the *spm23_A* gene was susceptible to SpM23_A, but all displayed diverse susceptibility toward SpM23_B, which gene was not identified in their genomes. The Vasteras_18:1 strain was the most susceptible to SpM23_B and thus was distinct from other Vasteras isolates. This is in agreement with phylogenetic analysis, which groups this strain in a distinct branch (Mansson et al., 2017).

Interestingly, we observed that whenever SpM23_A was efficient at lysing a particular bacterial strain, SpM23_B activity was diminished and vice versa. We hypothesize that this “mutually exclusive” resistance strategy could be based on the feature that most prominently differentiates the enzymes, namely, the surface electrostatic potential, which is reflected in their contrasting pI values. To test this, we performed a Cyt C assay, which allows for the evaluation of the net charge of the bacterial envelope based on the amount of positively charged cytochrome C binding. The assay results revealed differences in the surface charge of the tested *S. pettenkoferi* strains, with the DSM 19554 and VCU012 isolates having the most negative surface charge and Vasteras strains the most positive. The DSM 19554 strain with a negative surface charge was susceptible only to the SpM23_B enzyme and fully resistant to SpM23_A. In contrast, positively charged cells of Vasteras isolates were considerably more susceptible to SpM23_A than to SpM23_B. The susceptibility of *S. argensis* was the same as that of DSM 19954, with a net charge that lies at the midpoint between that of Vasteras and other strains (see also Supplementary Figure 5). Notably, strain VCU 012, which carries both genes, has a surface charge similar to that of DSM 19554, but is not cleaved by either enzyme. Overall, this experiment showed that there is an interplay between the bacterial surface charge and the charge on the enzyme surface and this link is reflected in lytic efficiency. Meanwhile, that the VCU 012 strain was resistant to both enzymes suggests that other mechanisms are involved in susceptibility-related enzyme-cell wall interactions.

#### CBD Binding

SH3b-type CBDs were previously shown to enhance bacterial cell lysis through increasing the affinity of the catalytic domain by anchoring it to its substrate (Grundling and Schneewind, 2006; Mitkowski et al., 2019). Here, we tested whether the lytic and binding spectra of SpM23_A and SpM23_B overlap by performing a CBD_A/CBD_B fluorescence binding assay in which recombinant CBD_A and CBD_B fused to GFP targeted the same set of closely related strains, as in the lytic assay (Figure 3B). The results showed that CBD_A binding specificity aligns well with the lytic activity of SpM23_A, meaning that, similar to that previously reported for SH3b domains, the CBD enhances the lytic activity of the M23 domain by placing it in physical proximity to its substrate. Surprisingly, CBD_B does not follow such a pattern. CBD_B binds strains with similar efficiency, irrespectively of whether SpM23_B cleaves (e.g., V_18:1 strain) or does not cleave (e.g., V_17:8 strain) their cell walls. This indicates that CBD_B binds the bacterial envelope with lower specificity than does CBD_A.

#### The Activity of SpM23_A, but Not SpM23_B, Is Observed in Bacterial Species

**Figure 4:**
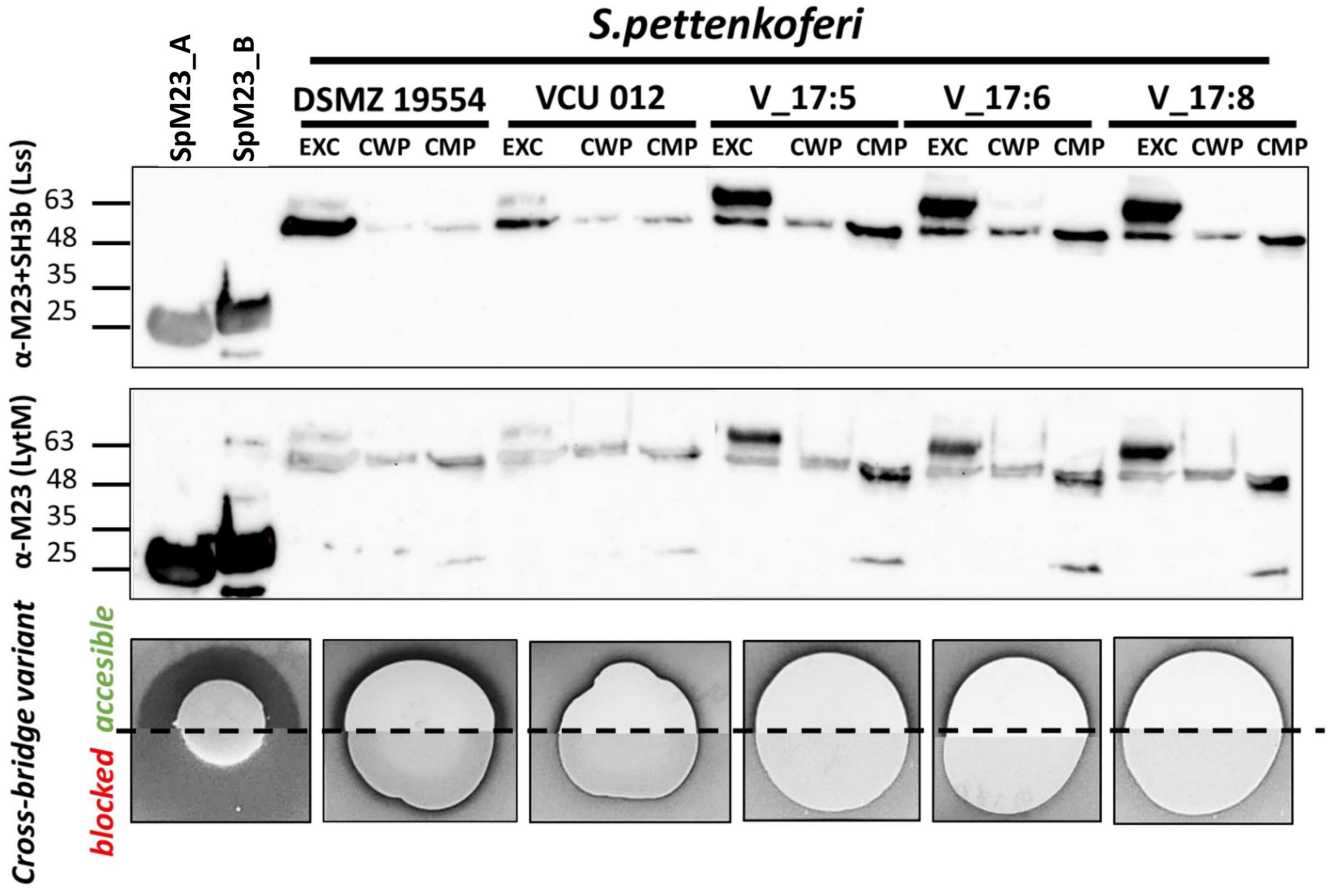
SpM23_A is produced under native conditions. Western blot analysis showing the presence of the SpM23_A protein identified in *Staphylococcus pettenkoferi* strains. EXC: extracellular fraction (concentrated 25-fold); CWP: LiCl-extracted cell wall protein fraction (concentrated 100-fold); CMP: SDS-extracted cell membrane protein fraction (concentrated 100-fold). Bioactivity assay of SpM23_A produced by *S. pettenkoferi* strains or spotted solution of recombinant enzyme (0.1μg, positive control). A *S. aureus* ΔtagO lawn was used as the biomarker owing to its susceptibility to SpM23_A in conditions of high ionic strength, such as in TSB medium. For additional controls, see Supplementary Figure 6.

The presence of signal peptides in both enzymes indicates that they are directed outside the bacterial cell. Accordingly, we sought to identify the exact localization of the enzymes in three fractions—extracellular, cell wall, and cell membrane. For this, we isolated proteins from these three fractions and performed Western blotting using antibodies against both the catalytic and cell wall targeting domains (α-M23+SH3b) or the catalytic domain alone (α-M23). A signal corresponding to the size of the full-length SpM23_A protein (unprocessed, ~50kDa) was detected in all the tested extracellular fraction samples; however, a slightly shorter variant (~48kDa) was also present in all the samples, irrespective of the subcellular localization. The estimated mass of this band corresponded to the enzyme form without a transmembrane domain (processed, ~47kDa with cleavage upstream of residue C28). Although the identity of the protein present in the extracellular band was confirmed by trypsin cleavage and mass spectrometry, we could not precisely identify the N-terminal processing site. Overall, we concluded that *S. pettenkoferi* strains direct SpM23_A to the extracellular environment as a full-length protein, where it is subsequently processed/activated. No other band that could represent SpM23_B was found in the *S. pettenkoferi* VCU 012 strain, implying that SpM23_B was not produced by this strain under the conditions used in this study (Figure 4; Supplementary Figure 6).

That the extracellular fraction is 4-fold less concentrated than the cell wall and membrane fractions is a clear indication that the strongest signal is derived from the extracellular fraction. Consequently, we attempted to detect SpM23_A enzyme activity *in vivo* in the extracellular, secreted fraction of the selected *S. pettenkoferi* strains. In this assay, the presence of the active enzyme is manifested as a clear zone around spotted bacterial cultures growing on a lawn of the *S. aureus* ΔtagO mutant. This mutant was selected for the *in vivo* studies owing to its susceptibility to SpM23_A because CBD_A displayed the highest binding efficiency toward this mutant (Supplementary Figure 4). As a control, we used the same mutant with the peptidoglycan cross-bridges (the substrate of the enzyme) blocked by preincubation with recombinant CBD_A. The experiment followed a previous assay performed with lysostaphin, where preincubation with CBD_Lss, and the consequent cross-bridge blocking in the targeted bacterial strain, led to a decrease in Lss lytic activity (data not shown).

*S. pettenkoferi* cultures spotted on a lawn of susceptible bacteria generated a clear halo, a direct demonstration of bacteriolytic activity. Because no lytic activity toward the substrate was observed when the cross-bridges were blocked by preincubation with CBD_A, we concluded that the clearing zones were the result of SpM23_A activity (Figure 4). The most prominent lytic area was observed with the *S. pettenkoferi* DSM 19554 strain and the least prominent with Vasteras isolates. It is tempting to link this to the high ratio of unprocessed to processed forms of SpM23_A observed with the Western blotting analysis, which hints that the Vasteras isolates produce full-length SpM23_A that display limited activity.

An analogous analysis was performed to detect the activity of SpM23_B; however, no clearing zones were observed with the SpM23_B-susceptible strain, *S. capitis* (Supplementary Figure 4). We also sought to induce the activity of this enzyme by challenging it with thermal stress, tunicamycin (a WTA synthesis inhibitor), pentaglycine excess, lysates of closely related strains, and milk, but without success (data not shown). This suggested that SpM23_B is either not produced, is produced in an inactive form, or the assay is not sensitive enough to detect SpM23_B activity in bacterial cells.

## Discussion

While screening for new M23 peptidoglycan hydrolases, we found two enzymes in one bacterial genome that shared modular structure and prominent sequence conservation, while also displaying strikingly different pIs. To our knowledge, this is the first report of the identification in same bacterial genome two so much alike peptidoglycan hydrolases, which display contrasting values in respect to their net charge.

This finding immediately raised a range of interesting questions, such as why one bacterium needs two homologous enzymes with such differing surface charges, what else differentiates these peptidoglycan hydrolases, what is their origin, and what are their possible physiological roles.

### SpM23_A and SpM23_B Are Lysostaphin-Type Peptidoglycan Hydrolases

Both enzymes have a multidomain architecture characteristic of many peptidoglycan hydrolases (Nelson et al., 2012; Vermassen et al., 2019). Their catalytic domains belong to the M23 family of metallopeptidases that are also found in lysostaphin, Ale-1, and zoocine A, and they are both linked to a cell wall-binding domain belonging to the SH3b family and also present in Lss and Ale-1 (Lu et al., 2006; Becker et al., 2009b). Although we did not determine which bond in peptidoglycan is cleaved by SpM23_A and SpM23_B, we demonstrated that both enzymes can lyse *S. aureus* cells. That M23 enzymes predominantly cleave peptidoglycan cross-bridges suggests that these are also substrates for SpM23_A and SpM23_B. It remains to be determined whether they cleave Gly–Gly bonds, as do lysostaphin and LytM, or D-Ala–L-Ala bonds, as occurs with zoocine or EnpA (Gargis et al., 2009b; de Roca et al., 2010).

Many, if not most, peptidoglycan hydrolases are produced as prepropeptides containing signal peptides and proregions. Whereas catalytic and cell wall-binding domains share high sequence homology among different peptidoglycan hydrolases, the proregions are very diverse. Nevertheless, these proregions still share some features. For instance, they generally contain repeated fragments, such as the 15 repeats of 13-amino-acid-long motifs present in the lysostaphin propeptide (Thumm and Gotz, 1997). In this study, we also found repeated sequences in both the characterized enzymes. Despite the large diversity in the sequences of propeptides found in peptidoglycan hydrolases, they have been shown to play the same inhibitory role. Indeed, many peptidoglycan hydrolases of diverse catalytic specificities are reported to be latent in their full-length form (Bublitz et al., 2009; Yang et al., 2012; Chao et al., 2013), including SpM23_A and SpM23_B. In the case of lysostaphin, a cysteine peptidase is responsible for the removal of the propeptide, leading to the activation of the enzyme (Thumm and Gotz, 1997), while for LytM, crystal structure analysis demonstrated a mechanism of inhibition based on blocking of the catalytic zinc coordination in the active site by additional residue and occlusion of the substrate-binding groove by part of the propeptide chain (Odintsov et al., 2004).

We have demonstrated *in vitro* that removal of the propeptide activates the SpM23_A enzyme. Interestingly, only a very small fraction of SpM23_A was found to be processed protein, with most being produced in its full-length or only slightly modified form. This is not surprising as the activity of peptidoglycan hydrolases has to be very tightly regulated to avoid uncontrolled cell lysis. Moreover, this agrees with the results of proteomic analysis of staphylococcal cell walls, where LytM is relatively abundant but only in its full-length, latent form (Pieper et al., 2006).

### Is SpM23_A an Autolysin?

Because several genetic and biochemical features of SpM23_A imply that it is involved in bacterial cell wall metabolism, we asked whether its function could be assigned to autolysis.

The *spm23_A* sequence is well conserved among *S. pettenkoferi* and is present in all strains of this species identified to date, indicative of a critical role. The lack of HGT hallmarks, a GC content close to the average for coding genes, and genetic neighborhood conservation all indicate that *spm23_A* was vertically inherited. Interestingly, an ABC transporter-encoding gene was identified close to *spm23_A*. This transporter displays similarities to the FtsEX complex, which is well conserved across the bacterial domain of life, and has been implicated in the regulation of a range of peptidoglycan hydrolases (Sham et al., 2011; Yang et al., 2011; Meisner et al., 2013; Mavrici et al., 2014). The FtsEX transporter complex is involved in peptidoglycan hydrolase activation following a conformational change, which leads to the induction of cell division (Pichoff et al., 2019). These observations provide additional evidence for the involvement of SpM23_A in daughter cell separation.

Numerous reports have demonstrated the importance of teichoic acids in the regulation of autolysins (Neuhaus and Baddiley, 2003), such as AltA, LytN, and Sle1 of *S. aureus* (Schlag et al., 2010; Frankel and Schneewind, 2012) and CD11 of *Clostridium difficile* (Wu et al., 2016). This is also the case for SpM23_A, which is much less active in the presence of teichoic acids (Supplementary Figure 4), a feature that can also be considered an additional indication of its autolytic function.

In agreement with its predicted localization, SpM23_A is directed to the extracellular environment predominantly as a propeptide (Figure 4). Proteolytic enzymes are frequently secreted as propeptides, a mechanism of protein activity regulation/control that has also been reported for autolysins; for instance, LytM is among the most abundant staphylococcal cell wall proteins, but only in its latent form (Pieper et al., 2006).

SpM23_A has not only been found in all tested *S. pettenkoferi* strains, but can also lyse all of them, albeit with different efficiencies. Lytic assays are commonly used for the evaluation of autolysin activity (Sabala et al., 2012; Raulinaitis et al., 2017; Sekiya et al., 2021). Recombinant autolytic enzymes or those isolated from native sources exert their activity by attaching to the cell walls of the producers and digesting their peptidoglycans, which leads to their lysis. Such activity has been demonstrated for numerous autolysins, including LytM and several other autolytic enzymes from *S. aureus*, such as LytN, Atl, and LytU (Odintsov et al., 2004; Biswas et al., 2006; Frankel et al., 2011; Osipovitch and Griswold, 2015; Raulinaitis et al., 2017). In contrast, bacteriocins do not usually act on host cell walls. The producer has to develop resistance to its bacteriocins based on modifications of peptidoglycan fragments targeted by these enzymes (Thumm and Gotz, 1997; Sugai et al., 1997a; Gargis et al., 2009a).

### Is SpM23_B a Bacteriocin?

In contrast to the *spm23_A* gene, the genetic distribution of *spm23_B* is limited to a few strains. This observation implies that SpM23_B is most likely redundant or plays an accessory role, suggesting that it may act as a bacteriocin. This idea is further supported by the clearly distinct genetic character of *spm23_B* and a set of functionally related genes that comprises the “*spm23_B* cluster,” indicating that its putative acquisition endowed certain strains with an adaptive advantage in their specific environmental niches.

Similar to other well-described bacteriocins, namely, Ale-1 and lysostaphin (Thumm and Gotz, 1997; Sugai et al., 1997b), *spm23_B* is located close to a gene involved in serine incorporation. This is suggestive of a possible resistance strategy against this hydrolase, an idea that was supported by lytic assays showing a lack of or reduced SpM23_B lytic activity toward the evaluated strains that harbor its coding gene.

### Surface Charge

The negative charge of SpM23_A is an intriguing feature. Most M23 peptidoglycan hydrolases and SH3b domains, such as Lss (Schindler and Schuhardt, 1964), LasA (Kessler et al., 1993), and Ale-1 (Sugai et al., 1997a), display high pIs resulting from basic surface charges.

The basic charge of peptidoglycan hydrolases was proposed to enhance the interactions between the proteins and negatively charged bacterial cell surfaces (Neuhaus and Baddiley, 2003; Weidenmaier and Peschel, 2008). Although this possibility has been challenged by several research groups, the outcomes have often been contradictory, leaving this question unanswered. Low et al. (2011) increased the activity of the isolated catalytic domain of endolysin by changing the charge from negative to positive. The observed effect was explained by enhanced interactions between the negative charge of the secondary polymers of the cell wall and the positively charged protein surface (Low et al., 2011). The same authors also demonstrated the opposite effect; i.e., enzyme activity was inhibited when the positive charge on the enzyme surface was inverted to a negative one. Similar effects of alternating net charge were also observed for cell wall-binding domains (Diez-Martinez et al., 2013). Here, we did not engineer the net charge of the enzymes, but instead tested their activity on bacterial strains of the same species (*S. pettenkoferi*) exhibiting different net surface charges as determined by Cyt C assay. The results revealed that there was a correlation between enzyme activity and bacterial surface charge. However, recently published results do not support Low’s hypothesis (Shang and Nelson, 2019), as an engineered increase in surface charge did not result in increased enzyme activity. These discrepancies may be explained by the presence of other regulatory mechanisms/elements.

The nature and role of charge in interactions between peptidoglycan hydrolases and bacterial cell walls remain unclear. To date, studies have mostly focused on engineering enzymes as a means of revealing these mechanisms, which always introduces some uncertainty regarding the structure and performance of the mutated proteins. The two enzymes reported here are naturally occurring and share the same modular architecture and very high amino acid sequence identity, while also displaying different surface charges. Accordingly, they might serve as an ideal model for studying the relationship between the charge on the surface of the bacterial cell wall and that on the surface of enzymes that cleave them.

### Conclusion

This work represents a comprehensive genetic and biochemical characterization of two novel members of the M23 family of peptidoglycan hydrolases. Despite being identified in the same bacterial host and sharing the same modular architecture and a high degree of amino acid sequence similarity, they display distinct pIs as well as genetic and biochemical features. Accordingly, we concluded that they play different roles in bacterial physiology.

## Data Availability Statement

The datasets presented in this study can be found in online repositories. The names of the repository/repositories and accession number(s) can be found in the article/Supplementary Material.

## Author Contributions

AW, EJ, and ŁŁ performed cloning and undertook the production, purification, and activity testing of the recombinant enzymes. AW performed the genetic and physiological analysis. AW, EJ, and IS analyzed the data and prepared the manuscript. EJ and IS provided the funding and supervised the project. All authors contributed to the article and approved the submitted version.

## Funding

The research was funded by the Foundation for Polish Science – FNP (PL) TEAMTech program (grant INFECTLESS – new generation of antibacterial wound dressing, POIR. 04.04.00-00-3D8D/16-00) and co-financed by the European Union under the European Regional Development Fund and grant Miniatura, 2017/01/X/NZ1/00512 of the National Science Centre, Poland.

## Conflict of Interest

The authors declare that the research was conducted in the absence of any commercial or financial relationships that could be construed as a potential conflict of interest.

## Publisher’s Note

All claims expressed in this article are solely those of the authors and do not necessarily represent those of their affiliated organizations, or those of the publisher, the editors and the reviewers. Any product that may be evaluated in this article, or claim that may be made by its manufacturer, is not guaranteed or endorsed by the publisher.

## Footnotes

^1^ https://img.jgi.doe.gov
