# Supplementary material for: Two New M23 Peptidoglycan Hydrolases With Distinct Net Charge
Source: Front Microbiol. 2021 Sep 24;12:719689. doi: 10.3389/fmicb.2021.719689 (PMC8498115; doi:10.3389/fmicb.2021.719689)
Supplement: Supplementary file 1 [file Data_Sheet_1.pdf]

Supplementary material

Table S1. The list of tested bacterial strains.

Species	Strain reference number/isolate
Escherichia coli	TOP10
Escherichia coli	BL21 (DE3)
Staphylococcus aureus	NCTC 8325-4
*Staphylococcus aureus**	RN4220 (ΔtagO mutant)
Staphylococcus argensis	DSM 29875
Staphylococcus capitis	PCM 2121
Staphylococcus pettenkoferi	DSM 19554
*Staphylococcus pettenkoferi***	VCU 012
*Staphylococcus pettenkoferi****	Vasteras_17:1 (V_17:1)
*Staphylococcus pettenkoferi****	Vasteras_17:2 (V_17:2)
*Staphylococcus pettenkoferi****	Vasteras_17:3 (V_17:3)
*Staphylococcus pettenkofer****	Vasteras_17:4 (V_17:4)
*Staphylococcus pettenkoferi****	Vasteras_17:5 (V_17:5)
*Staphylococcus pettenkoferi****	Vasteras_17:6 (V_17:5)
*Staphylococcus pettenkoferi****	Vasteras_17:7 (V_17:7)
*Staphylococcus pettenkoferi****	Vasteras_17:8 (V_17:8)
*Staphylococcus pettenkoferi****	Vasteras_18:1 (V_18:1)
Staphylococcus saprophyticus	PCM 2109
Staphylococcus simulans	CCM 3583

* kind gift of the Department of Infection Biology, University of Tübingen
** kind gift of the University of Virginia, USA
*** environment isolates, kind gift from Department of Orthopedics, Central Hospital Vasteras, Sweden (Mansson et al. 2017).

Table S2. List of the constructs and purified proteins used in this study.

Expression vector	Produced protein variant, abbreviation	Fused tag	Region, *length* [aa]	Theoretical pI	Mass [Da]
pMCSG7[1]	Full-length form, M23_A full-length	N-terminal his-tag, TEV protease cleavage site	1-457, *461*	4.33	49829.32
pMCSG7[1]	Length variant, $M23\_A_{190-457}$	N-terminal his-tag, TEV protease cleavage site	190-457, *267*	5.02	29182.78
pMCSG7[1]	Length variant, $M23\_A_{199-457}$	N-terminal his-tag, TEV protease cleavage site	199-457, *258*	5.53	28136.77
pMCSG7[1]	Length variant, $M23\_A_{209-457}$	N-terminal his-tag, TEV protease cleavage site	209-457, *248*	5.69	27379.01
pMCSG7[1]	Length variant, $M23\_A_{213-457}$	N-terminal his-tag, TEV protease cleavage site	213-457, *244*	5.86	26551.06
pET22b	Mature form, M23_A	-	217-457, *240*	5.68	26455.96
pET22b	Cell wall targeting domain, CBD_A	-	365-457, *93*	6.10	10524.68
pWALDO[2]	Cell wall targeting domain fused to GFP, CBD_A N-N-D-M	C-terminal GFP and his-tag	365-457, *351*	6.38	39601.30
pWALDO[2]	Cell wall targeting domain fused to GFP, CBD_A D-N-D-M	C-terminal GFP and his-tag	365-457, *351*	6.28	39620.32
pWALDO[2]	Cell wall targeting domain fused to GFP, CBD_A D-T-E-I	C-terminal GFP and his-tag	365-457, *351*	6.29	39603.32
pET22b	Mature form, M23_B	-	199-446, *248*	10.25	27194.00

[1]Stols, L., Gu, M., Dieckman, L., Raffen, R., Collart, F. R., & Donnelly, M. I. (2002). A new vector for high-throughput, ligation-independent cloning encoding a tobacco etch virus protease cleavage site. *Protein expression and purification*, *25*(1), 8–15. https://doi.org/10.1006/prep.2001.1603

[2]Waldo, G. S., Standish, B. M., Berendzen, J., & Terwilliger, T. C. (1999). Rapid protein-folding assay using green fluorescent protein. *Nature biotechnology*, *17*(7), 691–695. https://doi.org/10.1038/10904

Table S3. Comparison of amino acid sequence identity and similarity between selected parts of the M23 family enzymes. Numbers marked in **black** correspond to the alignments of the proregion, the **red** to the mature form of the enzymes. The background shades of grey represents the sequence similarity in the decreasing order. The values corresponds to percentage of **identity (bold)** and similarity (in parenthesis) calculated in Blastp algorithm (Altschul et al., 1990).

<table>
<tr><td colspan="2"></td><td colspan="3">Aligned aa sequence</td></tr>
<tr><td colspan="2"></td><td>Lss</td><td>Ale-1</td><td>M23_B</td></tr>
<tr><td rowspan="2">Aligned aa sequence</td><td>M23_A</td><td>29 (42)
60 (73)</td><td>26 (52)
59 (72)</td><td>34 (49)
65 (79)</td></tr>
<tr><td>M23_B</td><td>50 (62)
60 (75)</td><td>26 (53)
59 (73)</td><td>-</td></tr>
</table>

Table S4. Comparison of amino acid sequence identity and similarity between identified aminoacyltransferases neighbouring *m23_B* and its homologs. The background shades of grey represents the sequence similarity in the decreasing order. The values corresponds to percentage of **identity (bold)** and similarity (in parenthesis) calculated in Blastp algorithm (Altschul et al., 1990). The Uniprot numbers used for alignment are: aminoacyltransferase from *S.argensis* - WP_145115830.1; from *S.pettenkoferi* VCU 012 - EHM72111.1 and EHM72136.1; fmhA - WP_001012222.1; epr - WP_100495228.1; lif - WP_172684372.1; femA - WP_000673309.1; femB - WP_000673098.1, femX - WP_000413865.1.

	**Serine incorporation**			**Glycine incorporation**		
	FmhA *S.aureus*	Epr *S.capitis*	Lif *S.simulans*	FemA *S.aureus*	FemB *S.aureus*	FemX, *S.aureus*
Aminoacyltransferase, *S.argensis*	**61** (76)	**61** (76)	**59** (76)	**40** (60)	**35** (55)	**24** (47)
Aminoacyltransferase, *S.pettenkoferi*	**65** (81)	**66** (80)	**64** (79)	**38** (60)	**38** (57)	**25** (48)

Table S5. Genetic neighborhood of *m23_B* gene. Predicted function of the elements comprising "*m23_B* cluster". Source of presented data is Conserved Domains (Lu et al., 2020) or other servers indicated in the comments

Function related features						
**Protein in *S.pettenkoferi*** (GenBank ID)	**Conserved domains**	**Domain position, E-value**	**Function**	**Signal peptide?**	**pI**	**Comment**
**Acetyltransferase** (ASE36561.1)	1. Peptidoglycan/LPS O-acetylase OafA/YrhL<br>2. YrhL-like subfamily of SGNH-hydrolases	1.16-369, 2.98e-64<br>2.446-595, 2.19e-56	1. transferring acyl groups other than amino-acyl groups (**transferase**)<br>2. family of lipases and esterases (**hydrolase**)	Yes, membrane protein	9.41	Domains frequently identified together, might be involved in lipid metabolism (InterPro)
**M23_B** (ASE36562.1)	1. Peptidase M23<br>2. SH3b domain	1.233-326, 3.61e-36<br>2.365-428, 2.94e-12	1. Gly-Gly **hydrolysis** (endopeptidase)<br>2. **Binding** to PG	Yes, YSIRK-type	9.36	-
**Aminoacyltransferase** (CEP67_04475, locus tag)	FmhA protein of FemAB family	6-408, 3.01e-147	Lipid II:glycine glycyl**transferase**	No	5.68	Peptidoglycan interpeptide bridge formation enzyme
**LysM peptidoglycan-binding domain-containing protein** (ASE36563.1)	1. Lysin Motif<br>2. Predicted chitinase	1.412-454, 4.79e-13<br>2.535-692, 8.50e-17	1. N-acetylglucosoamine **binding** domain<br>2.hydrolysis of beta-1,4- linked polysaccharides (**hydrolase**)	Yes, YSIRK-type	5.68	Buist et al., 2008
**Acetyltransferase** (ASE36564.1)	Same as for above homolog	1.15-386, 1.61e-70<br>2.457-606, 1.65e-58	Same as for above homolog	Same as for above homolog	6.48	Same as for above homolog
**LytR family transcriptional regulator** (CEP67_04490, locus_tag)	Anionic cell wall polymer biosynthesis enzyme, LytR-Cps2A-Psr (LCP) family	34-286, 1.31e-62	1. transferring WTA from their lipid-linked precursor to cell wall PG (**transferase**)<br>2. Cell envelope-related function transcriptional attenuator common domain (**transcription regulation**)	No, membrane protein	9.59	Involved in the WTA biosynthesis in Gram-positive bacteria, extracellular domain of putative membrane-bound proteins (Kawai et al., 2011, Eberhardt et al., 2012)
**LysM peptidoglycan binding domain containing protein** (ASE36565.1)	1.Lysin Motif (two times)<br>2. CHAP domain	1.412-454, 2.52e-13; 471-514, 1.09e-11<br>2.535-692, 8.50e-17	1. Same as for above homolog<br>2. hydrolysis of L-muramoyl-L-alanine or D-alanyl-glycyl residues (**amidase/endopeptidase**)	Yes, YSIRK-type	5.60	Buist et al., 2008

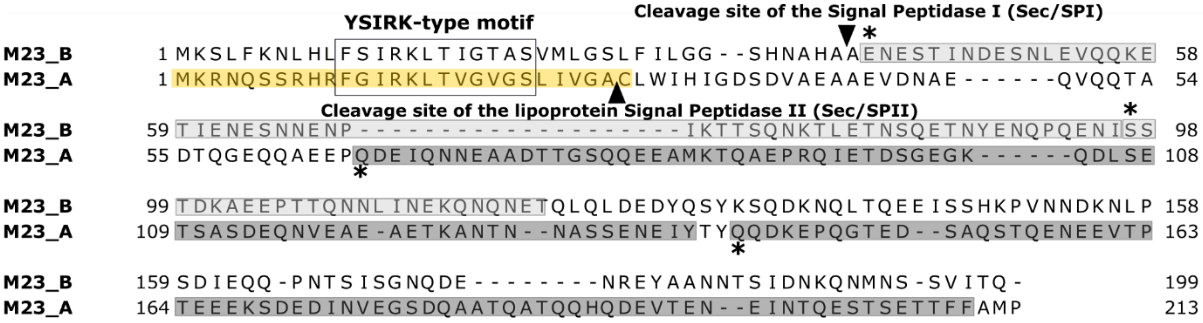

Figure S1. Sequence alignment of the M23_A and M23_B proregion sequences made in CLUSTAL Omega (Madeira et al., 2019). First amino acid of each predicted repeated sequence regions were indicated with asterix and whole region was highlighted in frames colored in the shades of grey. The prokaryotic lipoprotein lipid attachment profile was colored in yellow.

A

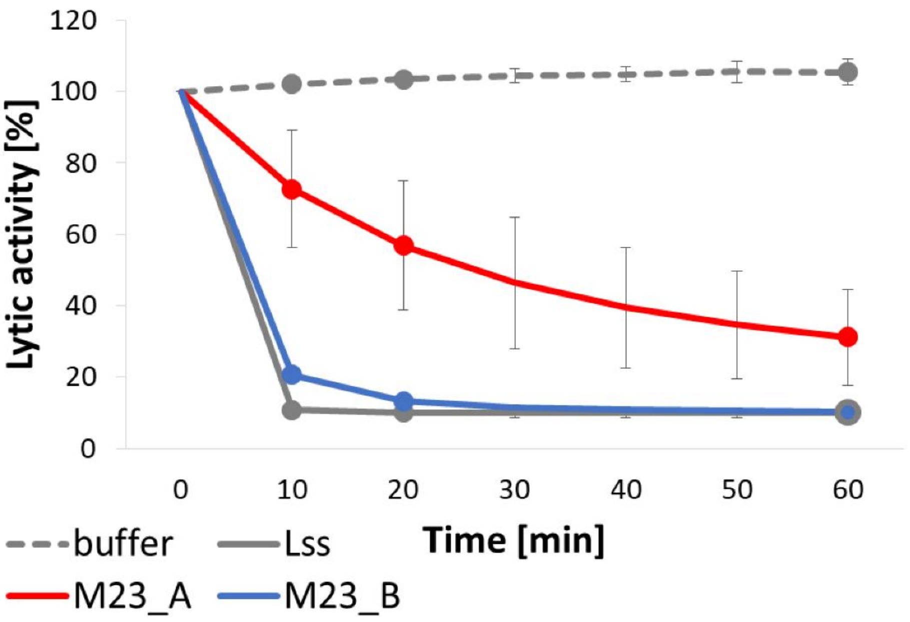

	buffer	Lss	M23_A	M23_B
Time [min]	Live cells (±SD) [cfu/ml], ***Eradicated cells [cfu/ml]***			
10	1.91 (±1.71) x10^8	1.49 (±1.04) x10^4 ***1.91 x10^8***	2.40 (±3.13) x10^8 -	2.07 (±1.45) x10^6 ***1.89 x10^8***
20	1.13 (±1.04) x10^8	1.50 (±2.34) x10^5 ***1.13 x10^8***	8.91 (±4.97) x10^7 ***2.42 x10^7***	5.62 (±4.96) x10^5 ***1.13 x10^8***
60	1.64 (±1.47) x10^8	3.24 (±3.55) x10^4 ***1.64 x10^8***	3.82 (±3.16) x10^7 ***1.26 x10^8***	5.67 (±5.92) x10^5 ***1.64 x10^8***

B

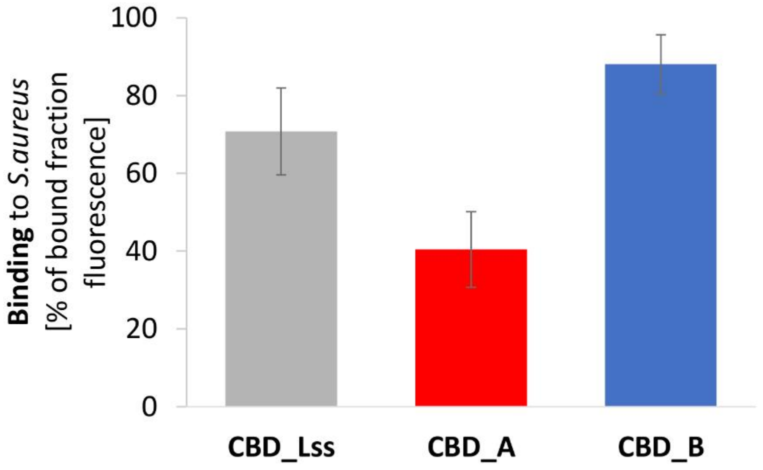

Figure S2. Lytic and binding assays of M23_A, M23_B and Lss against *S. aureus* NCTC 8325-4.

A. Bacterial cells eradication by M23_A, M23_B and lysostaphin. Around 10^8 CFU/ml of bacterial cells in 50mM glycine buffer was subjected to each enzyme (500nM) or the buffer alone and the decrease of OD_{620} was monitored every 10 minutes for 1 hour in room-temperature. At selected time points indicated on the chart with dots, the cells were plated on

the TSB-A in order to monitor live cells, the result of what was summarized in the table. Values presented on the chart are presented in relative [%] to the OD_{620} measured at time point zero. Values presented in the table show the live cells counted at each time point and the calculation of the eradicated bacteria in relative to the bacteria suspended in buffer at selected time point. The bars and the values in the table show the SD from three independent experiments.

B. Fluorescent binding assay showing the binding efficiency of the recombinant CBD derived from M23_A, M23_B or lysostaphin. The fluorescence of negative control (GFP alone) was subtracted from the presented results. The assay was performed in PBS buffer pH 7.2 in RT. The fluorescence was measured upon excitation set at 488nm and emission at 508nm wavelength.

A

	Position in aa sequence							
CBD_A sequence variant	**443**	444	**445**	446	**447**	448	**449**	Summarised frequency
N-N-D-M	N (64)	R (100)	N (64)	T (100)	D (85)	Q (100)	M (85)	598
D-N-D-M	D (36)	R	N	T	D	Q	M	570
D-T-E-I	D	R	T (36)	T	E (15)	Q	I (15)	402

B

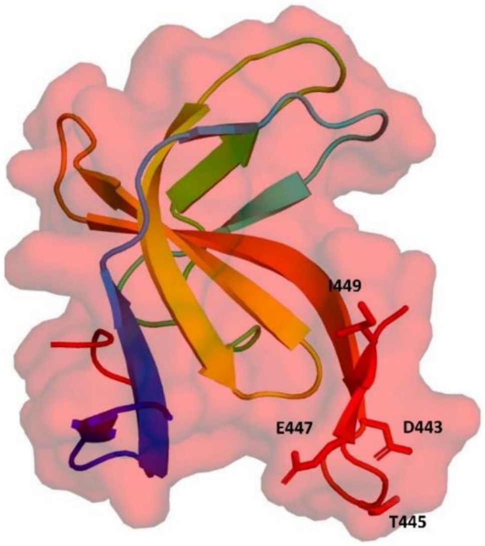

C

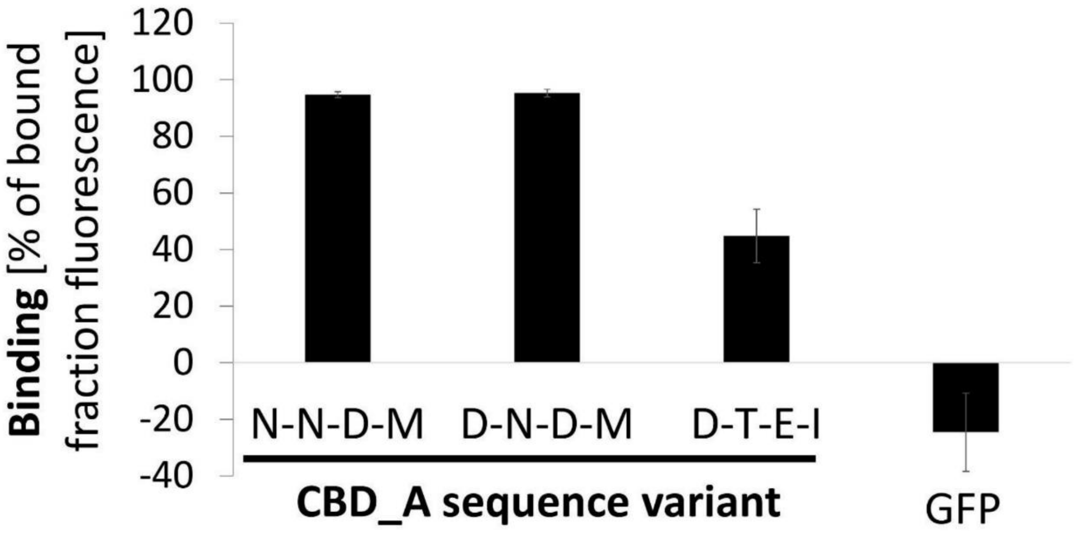

Figure S3. Comparative analysis of three CBD_A sequence variants identified in *Staphylococcus* spp. Variants were named according to the variable amino acid sequence.

A. Analysis of CBD_A sequence variation. In **bold** the variable positions are indicated. In parenthesis the occurrence frequency for each residue is indicated. Frequency of less common amino acid is highlighted in red. Summarised frequency score indicates the frequency of each variant occurrence. All listed CBD_A variants were subjected to binding assay.

B. The model of CBD_A D-T-E-I variant presented in the cartoon and transparent surface (40%) representations (SWISS-Model, Waterhouse et al., 2018). The spectrum of colors indicates the secondary structures order. The N-terminal CBD_A is shown in blue and the C-terminal in red. The localization of variable residues is marked with their side chains represented as sticks.

C. Binding assay for CBD_A fused to GFP performed on *Staphylococcus aureus* RN4220 ΔtagO. This mutant was selected because CBD_A displayed the highest binding efficiency toward its cell wall. The assay was performed in PBS, pH 7.2, at room temperature. Bacterial cells were mixed with 1 μM of each fluorescently labeled CBD_A. Cells were pelleted and the fluorescence (excitation: 488 nm, emission: 508 nm) of the unbound fraction of each CBD_A was measured. Results are expressed relative to sample without the addition of bacterial cells. The fluorescence of the negative control (GFP without CBD_A) is indicated on the chart as "GFP".

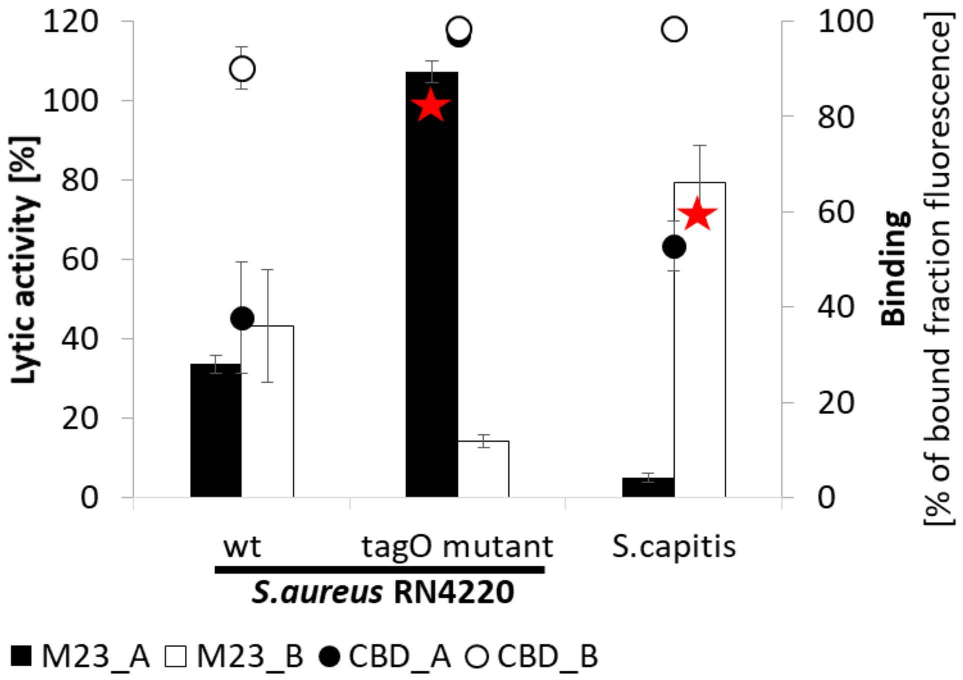

Figure S4. Lytic and binding assay performed in order to select the *Staphyloccus* species suitable for the binding and *in vivo* bioactivity assay. Susceptible *S. aureus* ΔtagO and *S. capitis* were lysed exclusively by M23_A or M23_B, thus were selected for the lawn formation for *in vivo* bioactivity assay as indicated with a red star. The lytic activity values indicated the OD_{600} decrease after 1h of reaction. The bacteria suspended in the reaction buffer without addition of the lytic enzyme were treated as negative control and the OD_{600} decrease values obtained for this variant were subtracted from the presented values. Assay was performed in 50mM glycine buffer pH 8.0, 100mM NaCl. The binding assay performed on the same species show the efficacy of binding of each recombinant CBD domain, indicative for that it could be used for the cross-bridge blocking. The binding assay was performed in PBS buffer pH 7.2 in RT. The fluorescence was measured upon excitation set at 488nm and emission at 508nm wavelength. The fluorescence of negative control (GFP alone) was subtracted from the presented results.

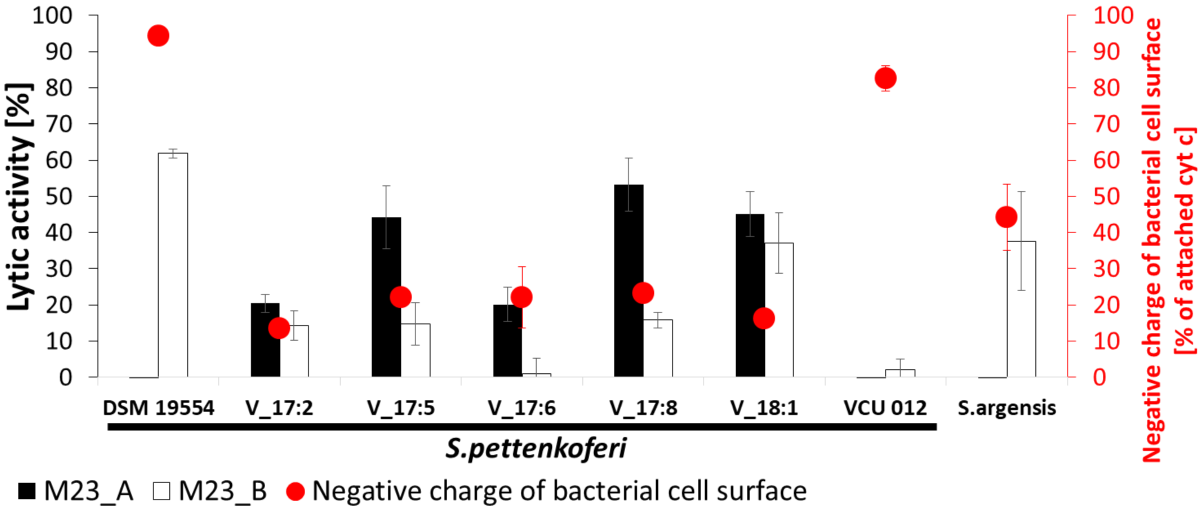

Figure S5. Lytic specificity of M23_A and M23_B toward host strains of their genes in M23_B buffer optimal conditions. Assay was performed in 50mM glycine buffer pH 8.0, 100mM NaCl. The lytic activity values indicated the OD_{600} decrease after 1h of reaction. The bacteria suspended in the reaction buffer without addition of the lytic enzyme were treated as negative control and the OD_{600} decrease values obtained for this variant were subtracted from the presented values. The red dots indicates the negative charge of each bacterial cell surface estimated in CytC assay, performed in the ammonium acetate buffer pH 4.6. All experiments were performed 3 times.

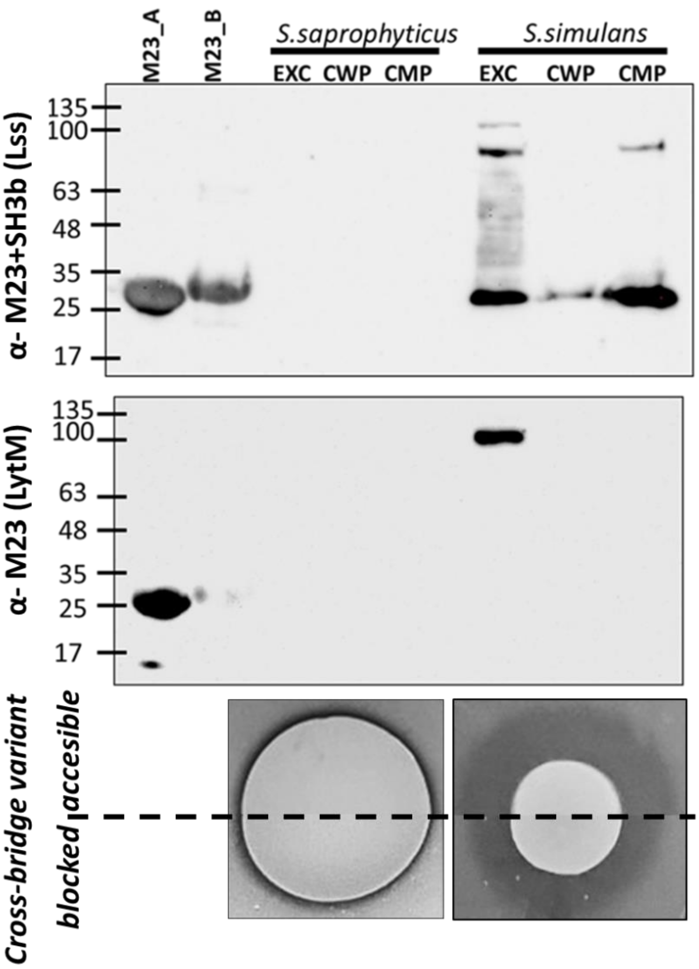

Figure S6. Western blot analysis investigating the cellular extracts of *S. saprophyticus* (close relative to *S. pettenkoferi*, negative control for M23_A production) and *S. simulans* (producer of the best characterized M23 enzyme, lysostaphin) serving as the control panel for analysis presented in **Figure 4**. EXC: extracellular fraction (concentrated 25-fold); CWP: LiCl-extracted cell wall protein fraction (concentrated 100-fold); CMP: SDS-extracted cell membrane protein fraction (concentrated 100-fold). Bioactivity assay of respective bacterial cultures against same bacterial lawn, as was used in *S.pettenkoferi* bioactivity assay, namely *S. aureus* ΔtagO.
